# Acquired angiokeratoma circumscriptum with spider configuration and spontaneous regression

**DOI:** 10.1016/j.jdcr.2025.06.032

**Published:** 2025-07-07

**Authors:** Marina Yunoki, Shumpei Kondo, Masaki Otsuka, Yoshiki Tokura

**Affiliations:** a Department of Dermatology & Skin Oncology, Chutoen General Medical Center, Kakegawa, Shzuoka, Japan; b Allergic Disease Research Center, Chutoen General Medical Center, Kakegawa, Japan

**Keywords:** angiokeratoma, circumscriptum, spider, spontaneous regression

## Introduction

Angiokeratomas are a group of capillary vascular ectasias characterized by dilated, thin-walled vessels in the papillary dermis. These vascular changes are associated with compact hyperkeratosis, papillomatosis, and acanthosis with elongated rete ridges in the epidermis.^1^ Five distinct clinical presentations of angiokeratomas are proposed, including solitary or multiple angiokeratoma; angiokeratoma of Mibelli, characterized by pediatric onset and predilection sites of acral areas; angiokeratoma of Fordyce, represented by angiokeratoma scroti; angiokeratoma corporis diffusum or Fabry disease; and angiokeratoma circumscriptum or angiokeratoma corporis circumscriptum naeviforme.^1^, ^2^, ^3^ Angiokeratoma circumscriptum is the rarest form of these disorders.^2^^,^^4^ It usually affects females, and the predilection site is the unilateral lower limbs, but other locations have also been reported.^2^^,^^4^ Initial presentation is typically at birth as red-colored macules. After several years, the lesions evolve into dark-red to blue-black confluent papules or nodules with hyperkeratotic features.^2^^,^^4^

Since there have been reported a considerable number of unusual cases of angiokeratoma circumscriptum,^5^^,^^6^ different types of angiokeratoma in the onset age, sites, and clinical appearance may be included in this category. Here, we describe a case of angiokeratoma circumscriptum with extremely unusual features, including spider shape, late onset, rapid progression, and spontaneous regression, further documenting a wide variety of this benign vascular tumor.

## Case report

A 63-year-old woman was referred to us because of a reddish reticular lesion on her right shoulder. It arose within a couple of weeks before her visit. One month before its occurrence, she had a pain in the upper back and shoulder, but she never had heat therapy. Going back further, 30 years ago, she had a bilateral shoulder pain and received a low-frequency wave therapy and some other minor treatments. Moreover, 40 years ago, she had a verrucous lesion on the right shoulder, which was treated with electrodessication and the warty lesion never reappeared thereafter. She had mild psoriatic arthritis for 30 years.

**Fig 1:**
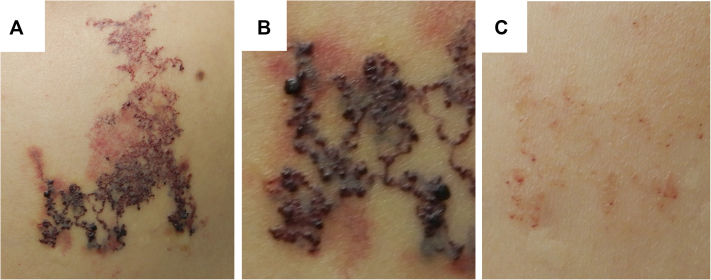
Clinical appearance. Irregularly shaped, *red* to *dark-brown* plaque **(A)**, with reticular telangiectatic vessels, radiating outward **(B)**. **C,** Spontaneously regressed lesion.

On our first examination, there was an irregularly shaped plaque, measuring 8.3 × 4.3 cm, on the right upper back to shoulder (Fig 1, *A*). It was an uneven, red to dark-brown lesion with reticular telangiectatic vessels, which partly merged together or radiated outward with tree-like appearance, forming spider configuration. On the radiating veins, there were multiple small papules, filled with blood (Fig 1, *B*). Ultrasonic echography disclosed dermal location of the lesion without extension to the deeper part. Complete blood counts were normal. Blood chemistries demonstrated that liver enzymes and renal values were within normal ranges and moderate hyperlipidemia was present.

**Fig 2:**
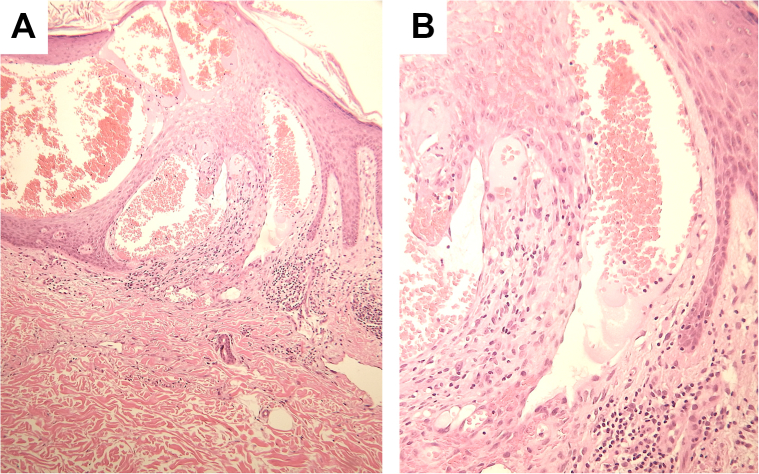
Histopathological findings. Ectatic capillaries in the papillary dermis beneath the epidermis **(A)**, and highly dilated subepidermal vessels lined by flattened endothelial cells **(B)**. Hematoxylin-eosin stain.

**Fig 3:**
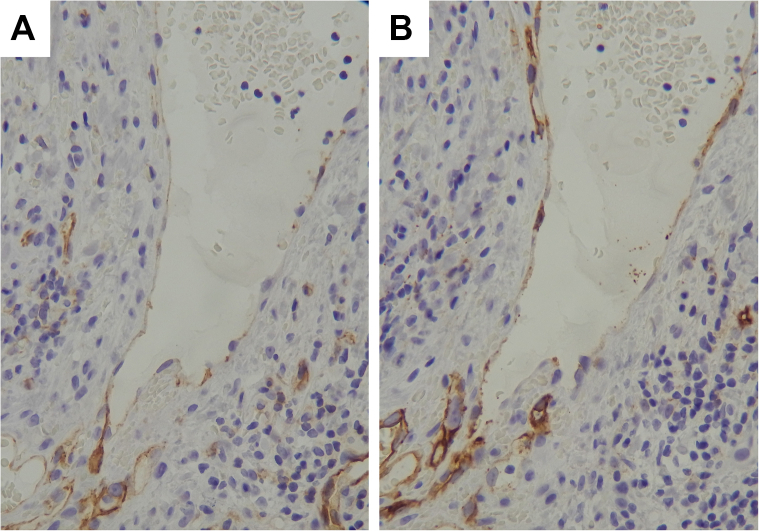
Immunohistochemical findings. Endothelial cells of dilated vessels are positive for CD31 **(A)** and only focally for D2-40 **(B)**.

A biopsy specimen revealed dilated and ectatic vascular channels in the papillary dermis beneath the epidermis (Fig 2, *A*). There were highly ectatic subepidermal vessels lined by flattened endothelial cells and filled with erythrocytes (Fig 2, *B*). Lymphocytes were scattered in the dermis. Notably, there were epidermal reactions, including acanthosis, hyperkeratosis, irregular papillomatosis, and hypergranulosis. Immunohistochemical staining revealed that vascular endothelial cells were positive for CD31 (Fig 3, *A*) and only focally for D2-40 (Fig 3, *B*), suggesting vascular derivation with weak lymphatic differentiation.^3^^,^^7^ We thus diagnosed the tumor as angiokeratoma cicumscriptum.

Two weeks after the biopsy, the lesion began to spontaneously involute and apparently regressed 2 months later (Fig 1, *C*). Eight months later, although the vascular lesion slightly recurred on the same site, it spontaneously subsided again without sequelae. Currently, 1 year after the initial development of the lesion, no recurrence was noted.

## Discussion

Among the five distinct types of angiokeratomas, angiokeratoma circumscriptum is the rarest condition.^2^^,^^4^ The documented clinical variations seem to give rise to some confusion in this category.^5^^,^^6^ Our case is characterized by spider configuration, acquired onset, rapid progression, and spontaneous regression. Especially, the clinical appearance in our case was different from the usual dark-red confluent papules or nodules commonly seen in angiokeratoma circumscriptum. It is considered that the initial event of angiokeratoma is capillary ectasia, followed by the epidermal changes. This scenario also may take place in our unique case.

Our patient had a long history of shoulder or back pain and received a low-frequency wave therapy and others. Although these treatments were performed a long time before, they might contribute to the long-standing background to trigger the proliferation or dilatation of capillary vessels. Several reports have shown the association of angiokeratoma with trauma.^8^^,^^9^ A case of angiokeratoma circumscriptum had a history of trauma preceding the onset of the lesion.^8^ In 3 patients, the telangiectatic lesions sharing the clinicopathological changes with angiokeratoma were induced by trauma.^9^ In 2 cases, thrombotic phenomena in angiokeratoma can induce spontaneous involution.^10^ Our case, together with these cases, suggests that some triggers, such as trauma or injury, stimulate capillary vessels beneath the epidermis to dilate, and after cessation of the stimuli, the lesions might regress.

The present case exhibited a bizarre clinical appearance and followed acquired onset and spontaneous regression. Accumulation of such cases is needed for further clarification of the incidence and possible causes in this type of angiokeratoma.

## Conflicts of interest

None disclosed.
